# Pinworm infection masquerading as colorectal liver metastasis

**DOI:** 10.1308/003588412X13373405384891

**Published:** 2012-09

**Authors:** KJ Roberts, S Hubscher, K Mangat, R Sutcliffe, R Marudanayagam

**Affiliations:** University Hospitals Birmingham NHS Foundation Trust,UK

**Keywords:** Liver, Misdiagnosis, Enterobius, Pinworms

## Abstract

*Enterobius vermicularis* is responsible for a variety of diseases but rarely affects the liver. Accurate characterisation of suspected liver metastases is essential to avoid unnecessary surgery. In the presented case, following a diagnosis of rectal cancer, a solitary liver nodule was diagnosed as a liver metastasis due to typical radiological features and subsequently resected. At pathological assessment, however, a necrotic nodule containing *E vermicularis *was identified. Solitary necrotic nodules of the liver are usually benign but misdiagnosed frequently as malignant due to radiological features. It is standard practice to diagnose colorectal liver metastases solely on radiological evidence. Without obtaining tissue prior to liver resection, misdiagnosis of solitary necrotic nodules of the liver will continue to occur.

Pinworm, *Enterobius vermicularis,* is considered the most common and least pathogenic of human helminthic infections.^1^ Residing primarily in the caecum, females migrate to the perineum nocturnally to lay eggs. Clinical associations are perineal discomfort and, less frequently, appendicitis or lower urinary tract symptoms in female patients.^2,3^ *E vermicularis *infections in the liver are extremely rare.^4^ The presented case involved liver resection for presumed colorectal liver metastasis (CRLM). Histological assessment, however, revealed an area of necrosis associated with *E vermicularis* infection.

## Case history

**Figure 1:**
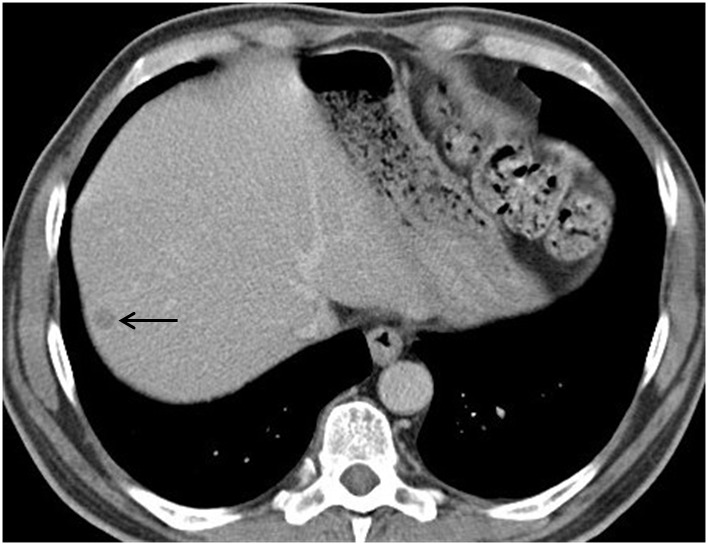
Contrast enhanced portal venous phase computed tomography demonstrating 2cm subcapsular lesion in segment 7 of the liver. The lesion displays typical low attenuation of a soft tissue mass characteristic of a colorectal liver metastasis.

A 44-year-old male with a 2-month history of rectal bleeding presented to his local hospital with abdominal pain and distension. He was a smoker, consumed moderate amounts of alcohol with a history of type 2 diabetes, hypertension and hypercholesterolaemia. Left-sided colonic obstruction was identified and at laparotomy a defunctioning colostomy was created proximal to a locally advanced rectal tumour (TNM stage T4NxMx). His staging computed tomography (CT) and magnetic resonance imaging (MRI) were reviewed at a dedicated hepatobiliary multidisciplinary meeting. A liver lesion consistent with a 2cm subcapsular CRLM was identified in segment 7 (Fig 1) with no further hepatic or extrahepatic disease.

Following completion of neoadjuvant chemoradiotherapy to downstage the pelvic disease, the patient underwent an uncomplicated non-anatomical liver resection. At this operation the liver was found to be macroscopically normal with the exception of the presumed CRLM. Intra-operative ultrasonography confirmed the presence of a hypoechoic lesion in keeping with appearances of a CRLM.

**Figure 2:**
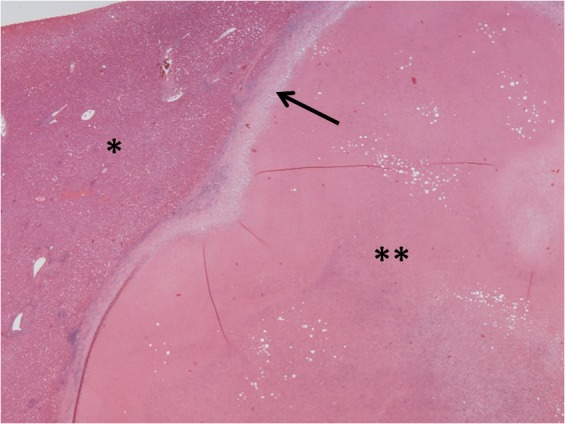
Haematoxylin and eosin stained section of normal liver (*) adjacent to the necrotic nodule (**) surrounded by a fibrous capsule (arrow)

**Figure 3:**
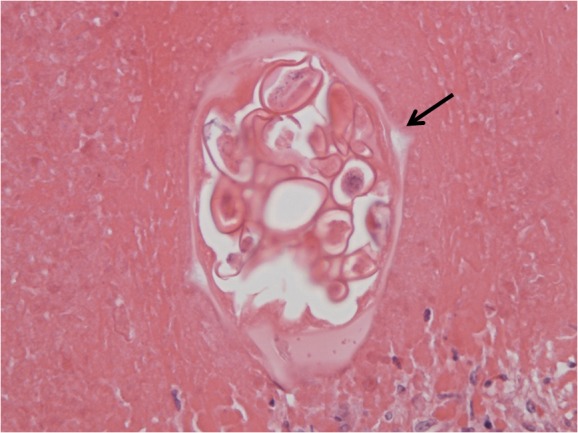
Haematoxylin and eosin stained cross-section of a female worm with its lateral alae (spine) marked by the arrow. Within the worm are several ova (one side concave, the other convex).

At pathological assessment, necrotic material was found in a fibrous capsule (Fig 2). The capsule was densely infiltrated by inflammatory cells, a high proportion of which were eosinophils. In the necrotic material multiple ova were observed, some of which were in a single worm (Fig 3). The ova are typical of *E vermicularis*, having both concave and convex surfaces. The specimen was excised completely with margins of normal liver. Prior to liver resection, the patient’s white cell and eosinophil count were normal (4.3 × 10^9^/1 and 0.2 × 10^9^/1; reference ranges: 3.5–9 × 10^9^/1 and 0–0.5 × 10^9^/1 respectively). There was no personal or family history of appendicectomy or perineal discomfort.

## Discussion

Liver resection for CRLM is the standard of care when possible.^5^ A diagnosis is made on a background colorectal adenocarcinoma supported by findings from CT and MRI. Obtaining tissue from the hepatic metastasis to confirm the diagnosis is not performed routinely due to risks of needle track seeding and the high sensitivity and specificity of radiological findings to identify CRLM correctly. Typical features of CRLM on CT during the portal venous phase are of hypodense lesions. On MRI, appearances can be variable but the lesions typically exhibit a moderately high signal on T2 weighted images. On T1 sequences following gadolinium, they are of lower signal than the background parenchyma on the portal venous phase and often have rim enhancement in the arterial phase. In the present case, these features were observed, thus appearing very much like a CRLM.

*E vermicularis* infection can result in a variety of diseases that typically affect the gastrointestinal tract and perineum. In female patients this can be manifested as lower urinary tract symptoms and in children/adolescents as perineal discomfort.^2^ *E vermicularis* is an accepted cause of appendiceal pain where infection can obstruct the lumen of the appendix, creating appendiceal colic.^3^ Rarely, *E vermicularis* is found outside of the gastrointestinal tract in the peritoneum, fallopian tubes and liver.^4,6,7^ It is suggested that a breach in intestinal continuity permits translocation of the parasite out of the intestine.

The patient in the presented case had no history of an appendicectomy or perineal discomfort. The rectal tumour was locally advanced (T4) and presumably provided a route of access out of the patient’s intestine, possibly spreading to the liver via the peritoneal cavity. The subcapsular location of the nodule supports this hypothesis. Previously published cases of *E vermicularis* liver infection are very limited but tend to be misdiagnosed as hepatic metastases in patients with established malignancy.^8^ Two of these cases followed a diagnosis of colorectal tumour,^9,10^ suggesting an association. However, it is possible that routine staging/surveillance cross-sectional imaging will identify these asymptomatic lesions and it may be that they are truly incidental as one case followed a diagnosis of cutaneous melanoma.^4^

The lesion described in this report demonstrates features consistent with the solitary necrotic nodule of the liver (SNNL). SNNL typically consist of central necrosis surrounded by fibrosis. These are uncommon lesions that are usually benign and often misdiagnosed as metastases.^11,12^ In a review of 51 cases of SNNL, features on CT mimic those of CRLM with lesions being hypodense and demonstrating peripheral enhancement with arterial or portal phases in nearly 40% of cases.^13^

## Conclusions

*E vermicularis* infection of the liver is exceptionally uncommon but has been reported previously following a history of colorectal carcinoma. It is unclear whether these asymptomatic liver lesions are being detected by coincidental cross-sectional imaging or whether there is a causal mechanism whereby the parasite may migrate from the intestine at the site of the tumour. The radiological features of *E vermicularis* infection in the liver mimic those of CRLM and of SNNL. For this reason, unless the fidelity or techniques of routine cross-sectional imaging change, benign liver lesions will continue to masquerade as metastases resulting in inappropriate intervention.
